# Explainable attention-enhanced heuristic paradigm for multi-view prognostic risk score development in hepatocellular carcinoma

**DOI:** 10.1007/s12072-025-10793-8

**Published:** 2025-03-16

**Authors:** Anran Liu, Jiang Zhang, Tong Li, Danyang Zheng, Yihong Ling, Lianghe Lu, Yuanpeng Zhang, Jing Cai

**Affiliations:** 1 Department of Health Technology and Informatics, Hong Kong Polytechnic University, 11 Yuk Choi Road, Hong Kong SAR, China; 2 Division of Computational & Data Sciences, Washington University in St. Louis, One Brookings Drive, St. Louis, MO 63130 USA; 3 Department of Anesthesiology, First Affiliated Hospital of Sun Yat-sen University, No. 58 Zhongshan Road 2, Guangzhou, 510060 Guangdong China; 4 Department of Pathology, Sun Yat-sen University Cancer Center, State Key Laboratory of Oncology in South China, 651 Dongfeng East Road, Guangzhou, 510060 Guangdong China; 5 Department of Liver Surgery, Sun Yat-sen University Cancer Center, State Key Laboratory of Oncology in South China, 651 Dongfeng East Road, Guangzhou, 510060 Guangdong China

**Keywords:** Hepatocellular carcinoma, Prognostic risk scoring, Deep learning-assisted diagnosis, Whole slide images, Necrosis, Lymphocytes, Interpretability, Disease-free survival, Overall survival, Artificial intelligence

## Abstract

****Purpose **:**

Existing prognostic staging systems depend on expensive manual extraction by pathologists, potentially overlooking latent patterns critical for prognosis, or use black-box deep learning models, limiting clinical acceptance. This study introduces a novel deep learning-assisted paradigm that complements existing approaches by generating interpretable, multi-view risk scores to stratify prognostic risk in hepatocellular carcinoma (HCC) patients.

****Methods **:**

510 HCC patients were enrolled in an internal dataset (SYSUCC) as training and validation cohorts to develop the Hybrid Deep Score (HDS). The Attention Activator (ATAT) was designed to heuristically identify tissues with high prognostic risk, and a multi-view risk-scoring system based on ATAT established HDS from microscopic to macroscopic levels. HDS was also validated on an external testing cohort (TCGA-LIHC) with 341 HCC patients. We assessed prognostic significance using Cox regression and the concordance index (c-index).

****Results **:**

The ATAT first heuristically identified regions where necrosis, lymphocytes, and tumor tissues converge, particularly focusing on their junctions in high-risk patients. From this, this study developed three independent risk factors: microscopic morphological, co-localization, and deep global indicators, which were concatenated and then input into a neural network to generate the final HDS for each patient. The HDS demonstrated competitive results with hazard ratios (HR) (HR 3.24, 95% confidence interval (CI) 1.91–5.43 in SYSUCC; HR 2.34, 95% CI 1.58–3.47 in TCGA-LIHC) and c-index values (0.751 in SYSUCC; 0.729 in TCGA-LIHC) for Disease-Free Survival (DFS). Furthermore, integrating HDS into existing clinical staging systems allows for more refined stratification, which enables the identification of potential high-risk patients within low-risk groups.

****Conclusion **:**

This novel paradigm, from identifying high-risk tissues to constructing prognostic risk scores, offers fresh insights into HCC research. Additionally, the integration of HDS complements the existing clinical staging system by facilitating more detailed stratification in DFS and Overall Survival (OS).

**Graphic Abstract:**

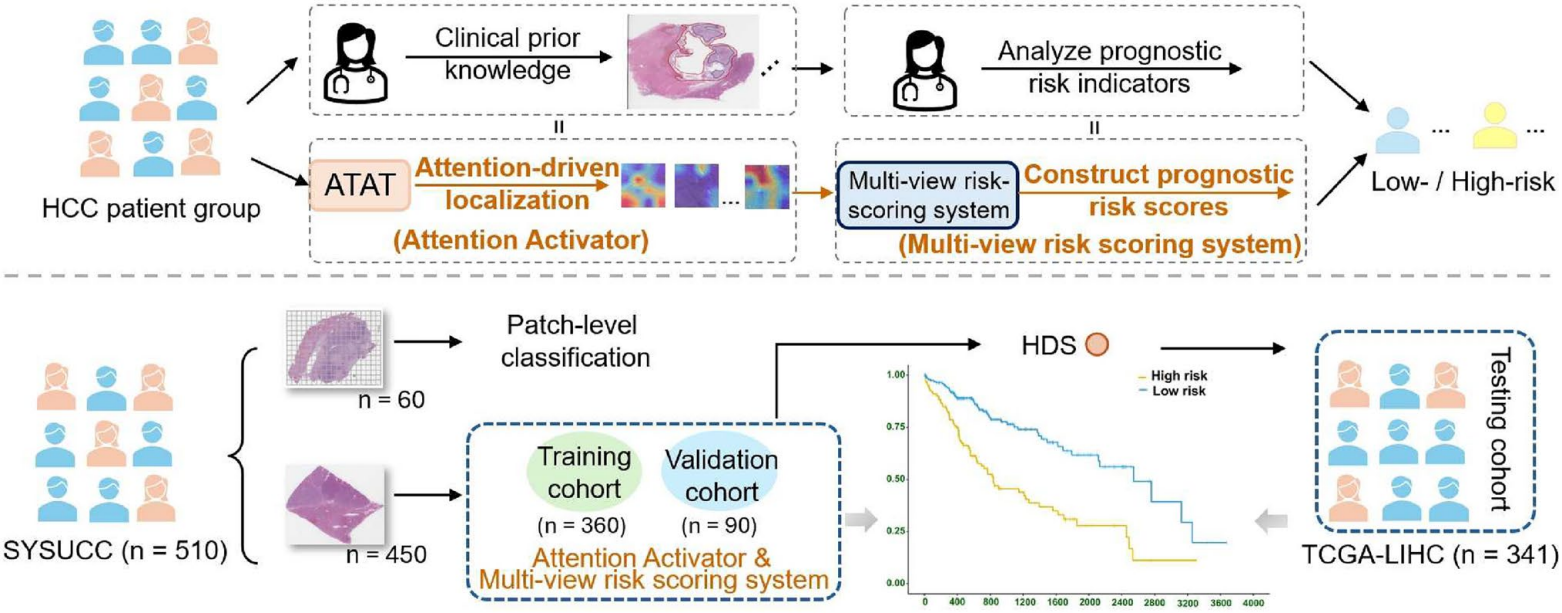

## Introduction

Hepatocellular carcinoma (HCC) is the sixth most common malignant tumor and the third leading cause of cancer-related mortality worldwide [1]. Common treatments for HCC include liver resection [2], liver transplantation [3], and immunotherapy [4]; however, not all patients benefit from these interventions [5]. This is because, despite the existence of several staging systems applied in clinical settings to help physicians develop individualized treatment plans for improving long-term prognosis, such as the Tumor-Node-Metastasis (TNM) [6] and the Barcelona Clinic Liver Cancer (BCLC) [7], prognostic variability persists within patients at the same clinical stage [8]. This variability often results in some patients being overtreated or undertreated. Efforts to refine these systems by incorporating more clinical factors have shown limited improvements in their accuracy or utility [9]. Thus, there is a pressing need to develop a new, more effective risk-scoring system for HCC to enhance prognostic assessments.

With advancements in computing and imaging technology, pathology has become crucial in cancer diagnosis and prognostic analysis [10, 11]. In particular, Whole Slide Image (WSI) analysis has benefited from deep learning applications in feature extraction, cancer diagnosis, and prognosis prediction, showing performance that often exceeds traditional clinical staging systems [12, 13]. However, many deep learning-based scoring systems face challenges in gaining clinical acceptance due to their lack of interpretability, which limits clinicians’ ability to understand and trust the predictions. For instance, black-box models that directly map WSIs to survival outcomes [14, 15] often fail to provide insights into which features or patterns drive the predictions, making clinical validation and integration difficult. Thus, a more explainable approach is to develop models that predict risk scores based on visually identifiable pathological risk factors, which can be independently validated by clinicians and correlated with established clinical staging systems.

In this study, we aim to establish a deep learning-assisted prognostic risk-scoring paradigm. We first identified tissue regions associated with high prognostic risk based on the attention-driven approach [16, 17]. This method focused on identifying key regions of interest (ROI) within WSI that contribute to the prognostic prediction. It allows the subsequent risk score construction to prioritize the pathological features within these ROIs. Therefore, we constructed three independent risk factors targeting microscopic, local, and macroscopic levels based on attention-derived ROIs. The Hybrid Deep Score (HDS) is obtained through a multi-view risk-scoring system to integrate these factors for predicting the prognostic risk in HCC patients.

## Method

### Patient cohort and study design

In this work, we collected the WSI corresponding to 510 patients diagnosed with HCC from Sun Yat-sen University Cancer Center (SYSUCC) as the in-house dataset. Among SYSUCC, 365 HCC patients were treated between March 2013 and December 2014 and 145 patients received treatment from August 2020 to June 2022. All patients and WSIs were one-to-one correspondence. The criteria for inclusion were as follows: (1) pathologically confirmed HCC; (2) absence of concurrent tumors; and (3) no metastases outside the liver. The exclusion criteria were as follows: (1) incomplete clinical data; (2) lack of high-quality WSIs; and (3) presence of other pathologic types of carcinoma within liver. Following the same criteria, we also selected 341 WSIs from the Cancer Genome Atlas Liver Hepatocellular Carcinoma dataset (TCGA-LIHC)^1^ as the external public dataset.

**Fig. 1:**
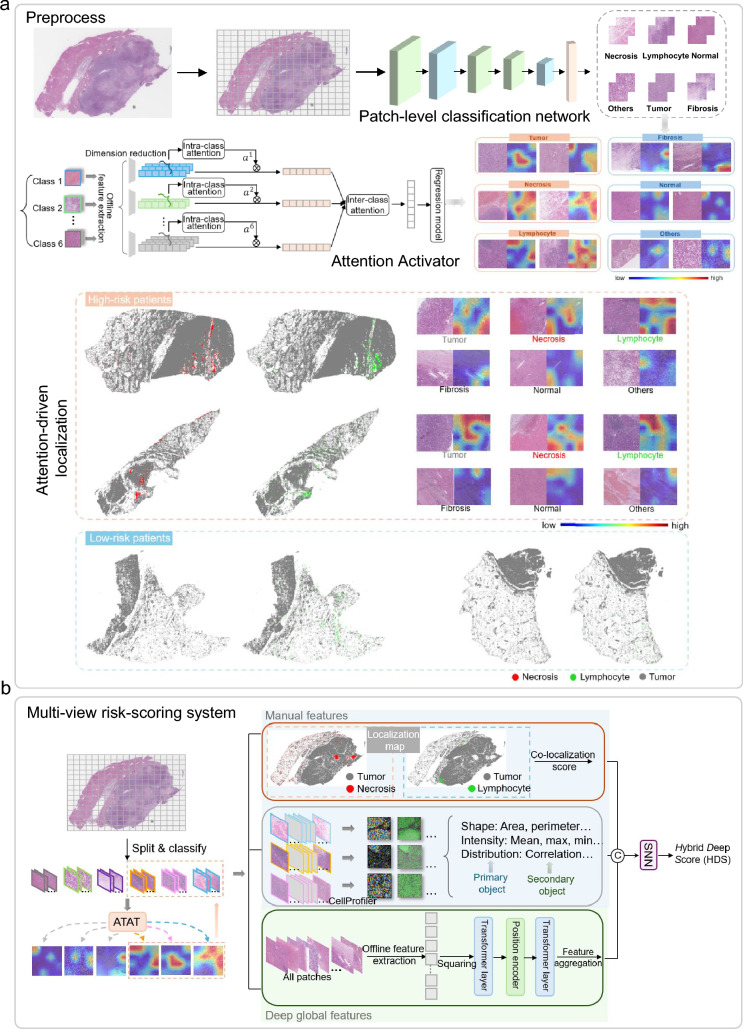
Workflow of patch-level classification network, ATAT, and Multi-view risk-scoring system. **a** WSIs are segmented into patches and categorized into six types using the pre-trained patch-level classification network, and then analyzed by ATAT which highlights potential tissue regions related to high-risk prognosis on a 16-patch grid. Localization maps and attention visualization reveal that in high-risk patients, WSIs typically show necrosis and lymphocytes near tumors, with ATAT emphasizing their junctions, whereas low-risk patients rarely exhibit these features. **b** Inspired by ATAT, microscopic morphological features, co-localization score, and deep global features are constructed and concatenated, then through an SNN, HDS is established. $\otimes$ weighted summation; *SNN* Self-Normalizing Neural Network; *ATAT* Attention Activator; $\textcircled {c}$ concatenation; the orange dashed box shows grids containing high-attention tissues

We randomly selected 60 WSIs from the SYSUCC, segmented them into patches, and annotated six distinct tissue categories-Tumor (1041), Necrosis (1017), Fibrosis (1007), Lymphocytes (1029), Normal Liver Cells (1041), and Others (such as blood vessels and steatosis, 994) for training a patch-level classification network. These categories are the most easily recognizable and representative tissues encountered. The HDS was developed using the remaining 450 WSIs in SYSUCC, utilizing an 8:2 training to validation split ratio, and tested on the TCGA-LIHC dataset to verify its generalizability across different treatment modalities. This study obtained ethical approval from the Institutional Review Board of Sun Yat-sen University Cancer Center and complied with the standards of the Declaration of Helsinki. All patients gave written informed consent for their archived tissue and clinical data to be used for scientific research in this study.

### Preparation and pre-process of WSIs

All samples were preserved using 4% neutral formaldehyde, embedded in paraffin, sectioned consecutively at a thickness of 4 ${\upmu }$m, and stained with hematoxylin and eosin (H&E). The 60 WSIs selected from the SYSUCC were segmented into 150 $\times$ 150-pixel patches at 20$\times$ magnification. We followed Clustering-constrained Attention Multiple instance learning (CLAM) [18] to remove patches with more than 75% blank areas.

Given the gigapixel of WSI, classifying and localizing each tissue patch in the WSI are crucial prerequisite steps for constructing new interpretable risk-scoring systems and performing prognostic analysis. PaSegNet [19] has shown promising performance in classifying liver pathological tissues. Inspired by this, we fine-tuned PaSegNet as our patch classification model to predict the labels and coordinates of each patch to localize the patches of the category of interest, as shown in Fig. 1a.

### Deep learning-based prognostic risk-related region discovery and risk-scoring system construction

To demonstrate the contributions of different tissue categories to prognostic prediction and support potential risk score construction, we designed ATAT, as illustrated in Fig. 1a. Patches labeled by a patch-level classification network were processed through an offline feature extractor (ResNet50 [20]), with features input into ATAT by category. ATAT assigns attention to each patch by modeling intra-class variability and inter-class relationships, allowing us to determine the prognostic significance of various tissue types. Local visualization results show that high-risk patients frequently exhibit necrotic areas and tumor-infiltrating lymphocytes. Further, ATAT predominantly focuses on junctions between necrotic, lymphatic, and tumorous tissues, while paying less attention to fibrotic and other areas. This motivates the construction of a risk-scoring system combining the influence of necrotic, lymphocyte, and tumor regions. Remarkably, the hypothesis generated by ATAT without prior experimentation was validated by experienced pathologists and aligned with conclusions from related studies [22–24].

To ensure clinical acceptability, we utilized the hypotheses based on ATAT as references to construct the interpretable risk score and verify its effectiveness in clinical practice. We developed the HDS, as shown in Fig. 1b, which models interactions between necrotic tissues, lymphocytes, and tumor regions. HDS captures information across three levels: 1) microscopic morphological features of three tissue types; 2) spatial interaction features (co-localization scores) between lymphocytes, necrosis, and tumor; 3) deep features representing global information from WSI. We utilized the DFS status and DFS time as the labels during the model training. Details on the structure of ATAT and the construction process of the HDS are provided in the supplementary materials.

### Statistical analysis

All statistical analysis was performed using R-software 4.0. The construction of HDS was carried out using PyTorch 1.12.0. Variables were compared using $\mathcal {X}^{2}$ test. Survival curves were generated with the Kaplan–Meier method and analyzed using the log-rank test. HRs with 95% CIs were estimated using univariable and multivariable Cox regression analyses. $p<0.05$ in a two-tailed test indicates statistical significance.

## Results

### Patient characteristics and clinical information

Online Resource 1, Table S1 presents the clinicopathological features of the SYSUCC cohort used to develop HDS, including the training cohort (n=360) and the validation cohort (n=90). There were no significant differences in baseline characteristics between the training and validation cohorts. Additionally, Online Resource 2, Table S2 provides the features of another 60 patients from SYSUCC, used for constructing a patch-level classification network. The patients in Table S2 have a similar distribution of clinicopathological features as those in Table S1. Furthermore, Online Resource 3, Table S3 details an external testing cohort of 341 patients from TCGA-LIHC. This cohort was not used during HDS development, allowing for an evaluation of HDS generalizability on unseen clinical data.

### Model evaluation and survival analysis

Ensuring the accurate classification of different tissue types by the patch-level classification network and the clinically meaningful localization of potential high-risk regions by ATAT are prerequisites for building an effective HDS. Online Resource 4, Fig. S1a shows that the average Area Under the Curve (AUC) across the six categories reached 0.98. Additionally, the confusion matrix in Fig. S1b further demonstrates the precision of the classification model in distinguishing between different tissue types. Further, ATAT has demonstrated a strong capability to distinguish between high- and low-risk patients (HR 2.77, 95% CI 2.04–3.74 in SYSUCC), as shown in Fig. S1c. It outperformed other state-of-the-art deep survival prediction models [14, 18, 25], achieving the c-index of 0.79 ± 0.035 (Fig. S1d). The series of findings related to high-risk patients in Fig. 1a are derived using ATAT. Since ATAT is a heuristic model and lacks clinical interpretability, we further designed a multi-view risk-scoring system and calculated HDS to enhance its clinical acceptability and applicability.

**Fig. 2:**
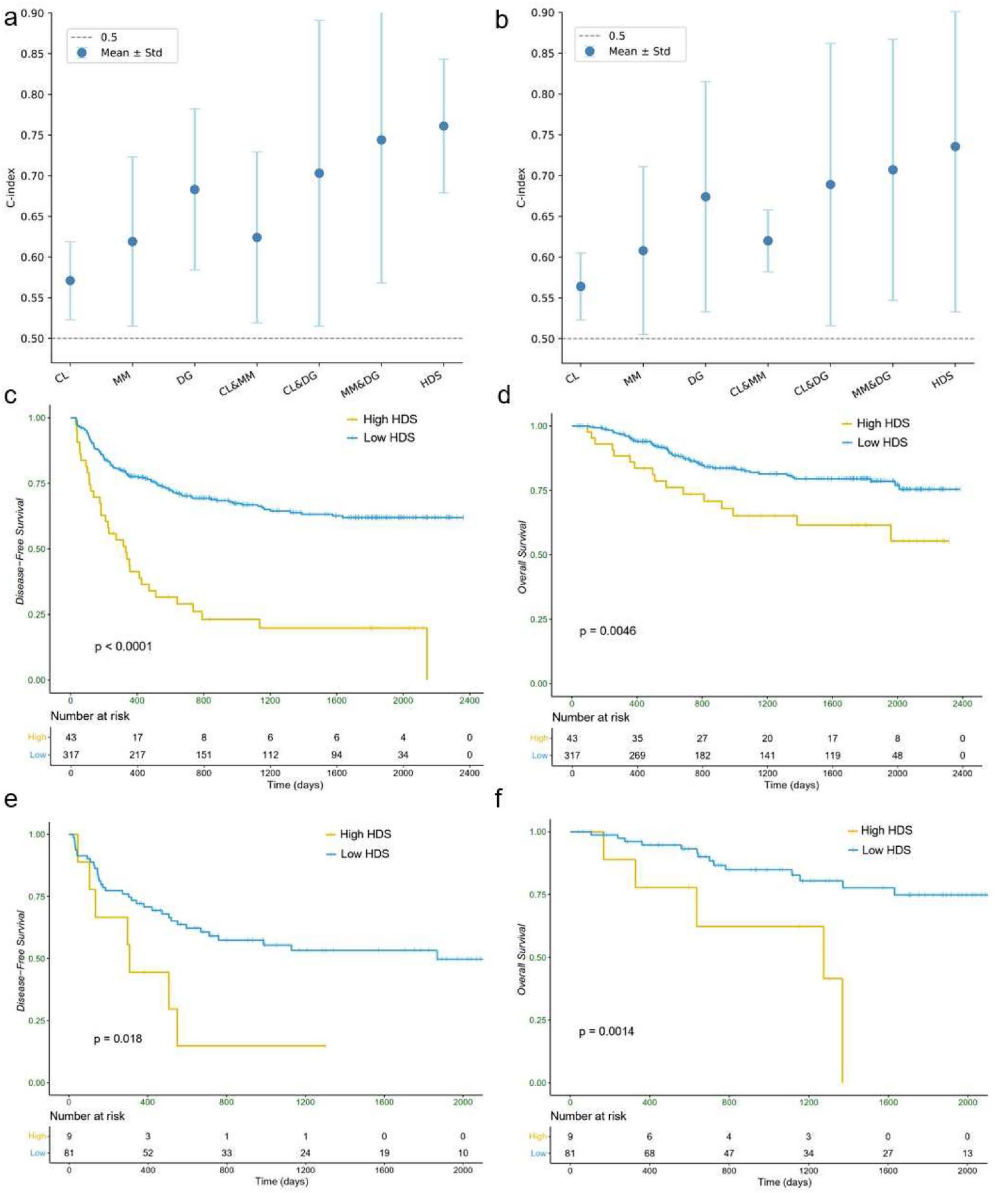
The constructed independent risk factors and their combinations from the three individual perspectives included in HDS and their c-index for DFS in SYSUCC **a** and TCGA-LIHC **b**. The Kaplan–Meier survival curves of HDS for DFS **c** and OS **d** in training cohort and validation cohort **e** and **f**. *MM* microscopic morphological features, *DG* deep global features, *CL* co-localization features, *HDS* hybrid deep score, *DFS* disease-free survival, *OS* overall survival

**Fig. 3:**
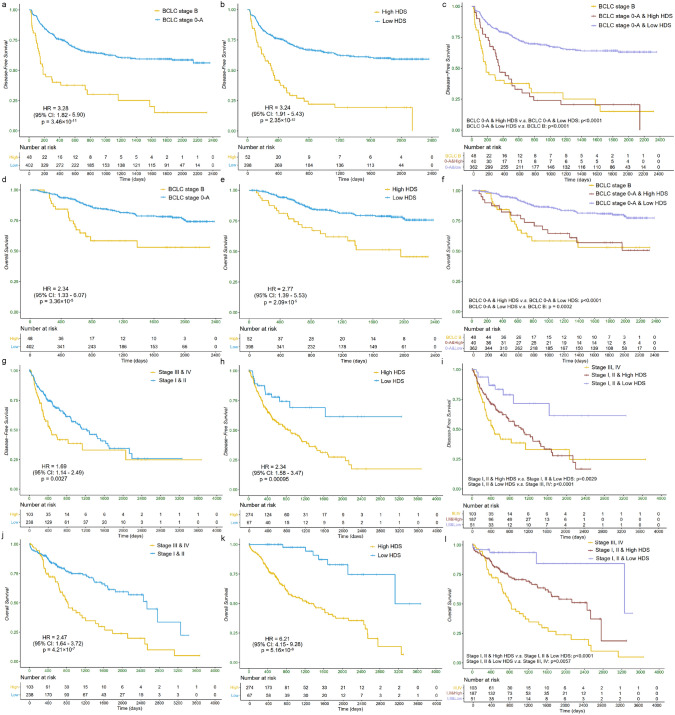
Kaplan–Meier curves for existing clinical staging systems and the refined stratification based on HDS in DFS and OS. **a** BCLC staging system, **b** HDS and **c** HDS-based refined stratification of BCLC stage 0-A of DFS in SYSUCC; **d** BCLC staging system, **e** HDS and **f** HDS-based refined stratification of BCLC stage 0-A of OS in SYSUCC; **g** TNM staging system, **h** HDS and **i** HDS-based refined stratification of TNM stage I&II of DFS in TCGA-LIHC; **j** TNM staging system, **k** HDS and **l** HDS-based refined stratification of TNM stage I&II of OS in TCGA-LIHC. *BCLC* Barcelona Clinic Liver Cancer, *TNM* American joint committee on cancer tumor node metastasis, *HDS* hybrid deep score, *DFS* disease-free survival, *OS* overall survival

In Fig. 2a, b, Online Resource 5 and 6 (Table S4a and S5a), we evaluated the c-indexes and 1-, 2-, and 5-year AUCs using single view or two of the three views of indicators (CL co-localization features, MM microscopic morphological features, DG deep global features & means concatenate two types of features) on SYSUCC and TCGA-LIHC. The experimental results demonstrate that combining any two types of features can further improve prognostic performance compared to using a single indicator alone. For instance, combining MM and DG resulted in a 20% increase in the c-index on SYSUCC compared to MM. Finally, integrating all three perspectives as HDS achieved more competitive performance (the AUCs of HDS for the 1-, 2-, and 5-year predictions and c-index of DFS were 0.682±0.183, 0.724±0.117 0.767±0.138 and 0.751±0.082 in SYSUCC and 0.678±0.238, 0.735±0.219, 0.723±0.130 and 0.729±0.196 in TCGA-LIHC). Furthermore, we have provided the results of the multivariable analysis of these three indicators of DFS on SYSUCC and TCGA-LIHC in Table S4b and Table S5b, and the multivariable analysis confirms that these three factors are independent prognostic risk factors (with multivariable $p<$0.001). Additionally, Table S4c and Table S5c present the pairwise $\mathcal {X}^{2}$ test results for these three indicators on the two datasets and the $\mathcal {X}^{2}$ $p>$0.05 indicate that the features from these three perspectives are mutually independent.

For HDS, the median value of the training cohort was taken as the cut-off value of high- and low-risk groups. Patients with a lower HDS experienced longer DFS and OS durations across both training and validation cohorts. Generally, as shown in Fig. 2c–f, the 2-year DFS rate in the high-risk group reached 26.79% in the training cohort and 16.22% in the validation cohort, compared to the low-risk group with DFS rates of 70.69% and 66.21% in the training and validation cohorts, respectively. Additionally, using OS as a secondary endpoint, HDS also effectively differentiated between high- and low-risk groups: the 2-year OS rate in the high-risk group was 69.24% in the training cohort and 60.31% in the validation cohort, compared to 87.21% and 86.49% in the low-risk group. In long-term (5-year) DFS and OS, HDS also demonstrated similarly superior risk stratification capabilities as seen in the short-term (1-/2-year) analysis.

Kaplan–Meier curves were constructed in Fig. 3 to illustrate the role of HDS in survival risk stratification. Building upon the existing BCLC and TNM staging systems, we further stratified the low-risk subgroups based on HDS. As shown in Fig. 3c, f, i, l, the *p* values between low-risk group with high HDS v.s. the low-risk group with low HDS were all less than 0.001 for both DFS and OS. This indicates that HDS can effectively identify potential high-risk patients within the low-risk groups, enabling clinicians to provide enhanced monitoring for these patients.

Additionally, in Online Resource 7, Table S6, we evaluated the prognostic performance of combining the existing clinical staging systems with HDS (use the linear combination of HDS and clinical staging as input for the Cox model). The results demonstrated that the combination of BCLC/TNM staging systems with HDS consistently achieved the best performance across both datasets (c-index: 0.783±0.110 in SYSUCC, 0.747±0.091 in TCGA-LIHC; 1-year AUC: 0.701±0.045 in SYSUCC, 0.681±0.060 in TCGA-LIHC; 2-year AUC: 0.739±0.213 in SYSUCC, 0.741±0.073 in TCGA-LIHC and 5-year AUC: 0.772±0.224 in SYSUCC, 0.740±0.208 in TCGA-LIHC). This further highlights the complementary role of HDS in improving the prognostic accuracy of existing clinical staging systems.

### Prognostic risk factors of HDS

Cox proportional hazards regression analysis was conducted to identify independent predictors for DFS in SYSUCC (Online Resource 8, Table S7) and TCGA-LIHC (Online Resource 9, Table S8). Since the known clinicopathological factors differed between the two cohorts, each was considered separately in the univariate Cox analysis. Subsequently, multivariable analysis revealed that tumor size (HR 3.275, 95% CI 2.053–5.219), tumor number (HR 1.541, 95% CI 1.326–4.358), and HDS (HR 3.852, 95% CI 2.333–5.319) were independent risk factors for DFS in SYSUCC, while in TCGA-LIHC, depth of invasion (HR 2.214, 95% CI 1.6–3.064) and HDS (HR 2.449, 95% CI 1.797–5.312) were identified as independent risk factors for DFS.

**Fig. 4:**
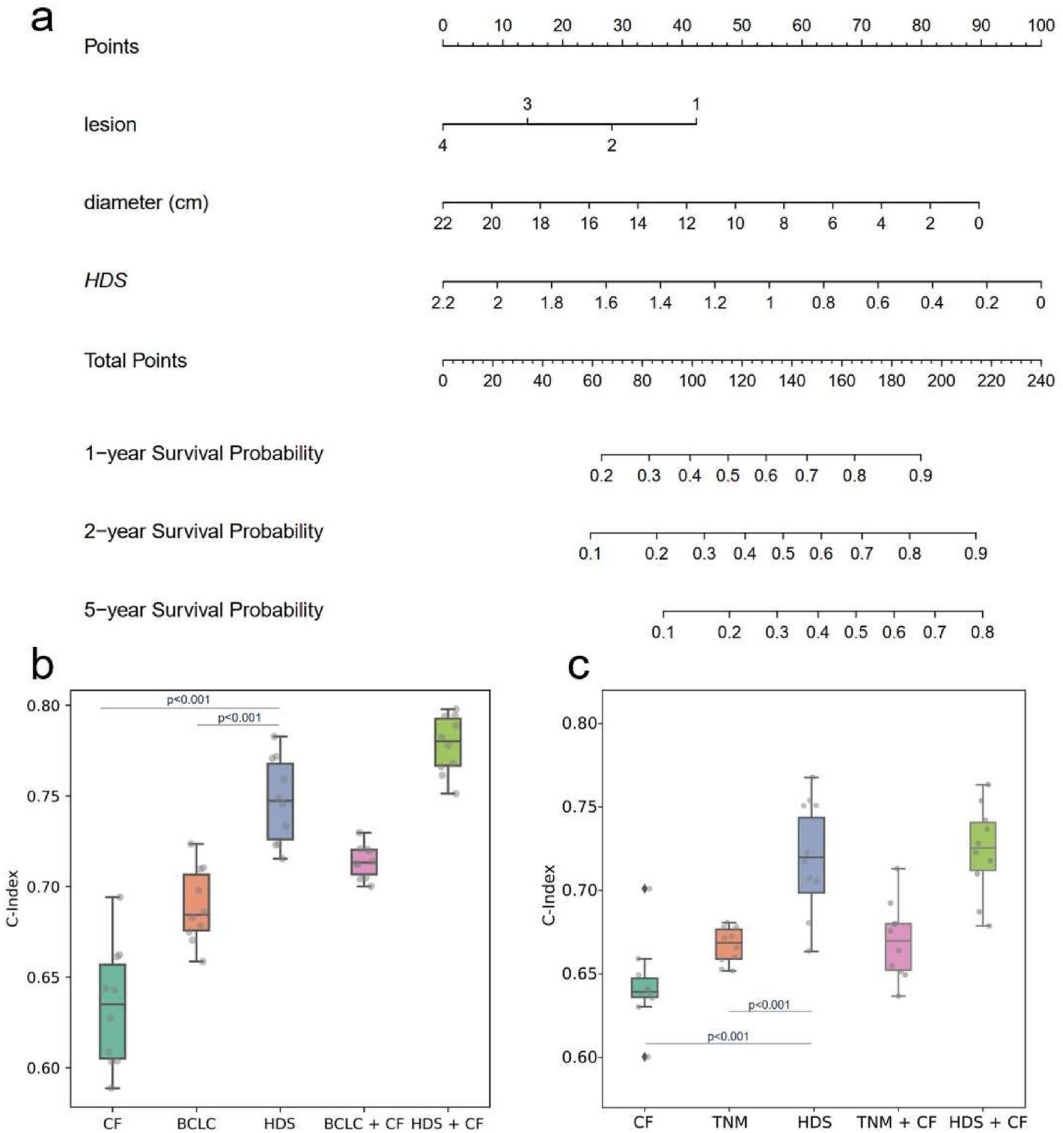
**a** Nomogram of independent clinical risk factors and HDS for DFS in SYSUCC. **b** C-indexes of independent CF, BCLC stage, HDS, and BCLC/HDS combined CF in SYSUCC. **c** C-indexes of independent CF, TNM stage, HDS, and TNM/HDS combined CF in TCGA-LIHC. *CF* clinical risk factors, *BCLC* Barcelona Clinic Liver Cancer, *TNM* American joint committee on cancer tumor node metastasis, *HDS* hybrid deep score, *DFS* disease-free survival

To incorporate these independent clinical risk factors (“Number of lesions" and “diameter of tumor") from the multivariable analysis, the nomogram was constructed for DFS in the SYSUCC cohort (Fig. 4a). Additionally, Fig. 4b presents the c-indexes for approaches considering only these clinical features, BCLC staging, and HDS. Notably, HDS achieved competitive performance (0.747, 0.711–0.783), and the integration of HDS with clinical features led to the improvement in predictive performance (with a 4.1% increase). In Fig. 4c, the combination of HDS with clinical features also demonstrated enhanced performance in TCGA-LIHC (showing a 2.87% increase). These results present the complementarity of HDS with clinical factors, offering valuable insights for augmenting and refining survival prediction methods based on existing clinical features.

**Fig. 5:**
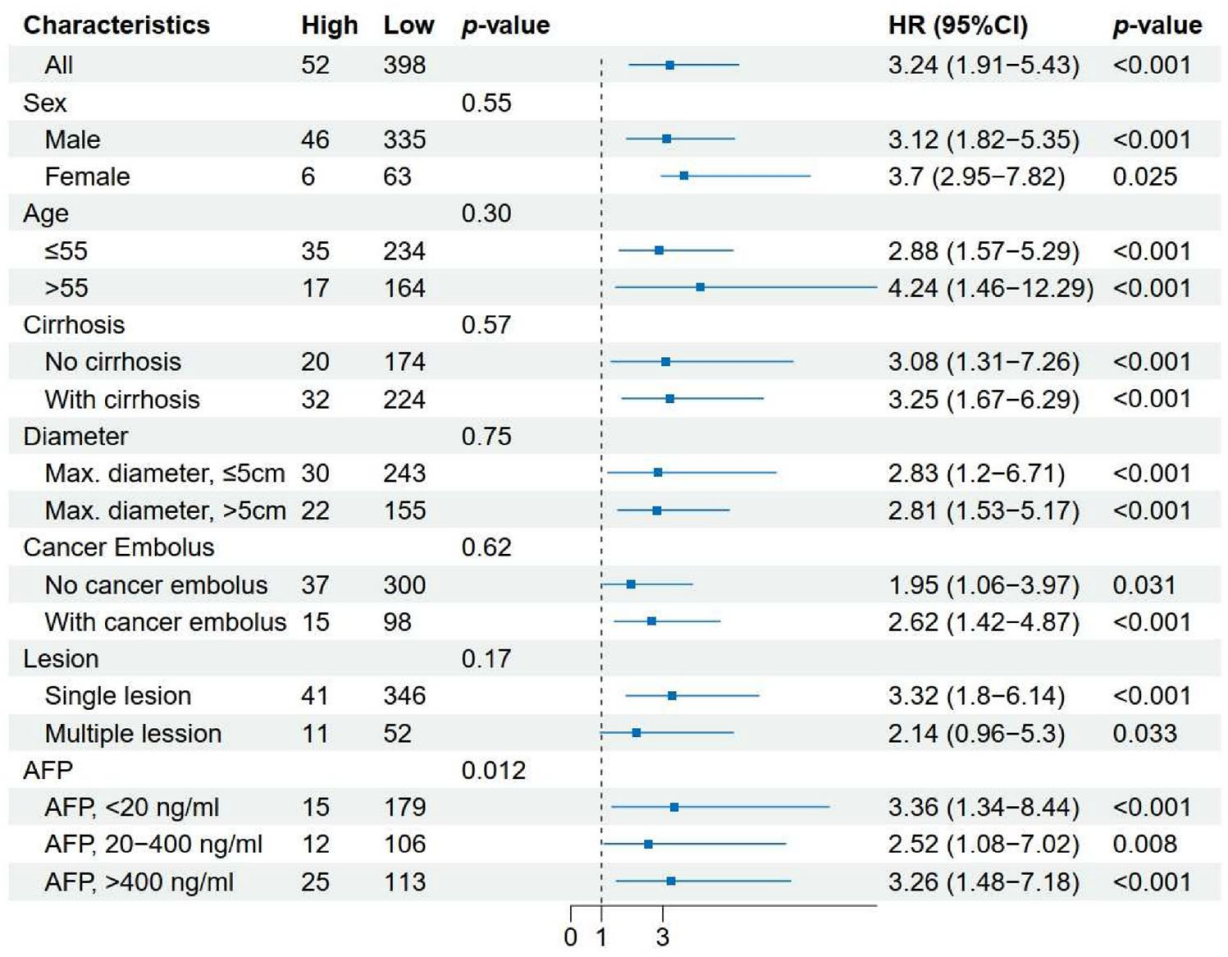
Forest plot of HDS for DFS in SYSUCC. *AFP* alpha-fetoprotein, *DFS* disease-free survival

The forest plot in Fig. 5 demonstrates that after stratifying the SYSUCC into different subgroups based on clinical characteristics, HDS remained an effective predictor for DFS with p < 0.05 in each subgroup. Similarly, the forest plot in Online Resource 10, Fig. S2 for the TCGA-LIHC cohort confirmed that HDS serves as a prognostic factor across all clinical subgroups.

## Discussion

As a common and highly lethal malignant tumor, it is of clinical significance to construct effective prognostic risk-scoring systems for HCC. Such systems can assist physicians in devising personalized treatment plans that potentially reduce the risk of recurrence and mortality for patients. While existing risk-scoring systems have advanced the field significantly, challenges remain in balancing interpretability and automation. For instance, manual feature extraction by pathologists, though highly informative, can be time-consuming, and entirely relying on black-box deep learning models may limit clinical interpretability. To address these challenges, we proposed a deep learning-assisted paradigm for constructing risk scoring based on WSI analysis and developed a novel prognostic risk score, named HDS.

In this study, following the general process employed by pathologists for constructing risk-scoring systems, we developed an attention-enhanced heuristic paradigm assisted by DL to generate prognostic risk scores. The DL-based assistance in this paradigm is manifested in two primary aspects: first, it identifies areas related to potential high-risk regions for physicians’ reference based on attention mechanisms enabling the simplified post-processing step; second, interpretable risk factors from three distinct perspectives are extracted and integrated by DL models. Utilizing this heuristic paradigm, we constructed HDS for HCC patients. The prognostic analysis results indicate that HDS not only demonstrates competitive performance in predicting the prognosis of HCC patients but most importantly complements and refines existing clinical staging systems (such as BCLC and TNM). Integrating HDS into existing prognostic clinical staging systems allows for the further identification of potential high-risk patients, thereby providing physicians with valuable references for developing more detailed tracking and treatment strategies.

In previous work, the localization of tissues associated with potential high-risk groups has often relied on prior knowledge of pathologists. For example, [26] focused on investigating the impact of mucus and stromal tissue on the prognosis of colon cancer, and [27] explored the relationship between aneuploidy and the prognosis of malignancy. However, a significant challenge of such approaches is their ineffectiveness when clinical priors are lacking, especially in the context of newly emerging cancers or rare cancer subtypes. Therefore, a heuristic approach is needed that can identify potential high-risk regions associated even in the absence of relevant clinical expertise. Thus, we first designed the ATAT to locate tissues of potential high-risk regions in WSIs. The attention values reflect the focus of ATAT on different tissue types during prognostic prediction. Unlike attribution map methods [19], whose results largely depend on model performance and where pixel-level feature attribution maps are not conducive to pathologists directly observing details in the original image, ATAT offers pathologists interpretable references for identifying potential risk-related areas.

On the other hand, while it is possible to locate tissue regions associated with high-risk prognostic groups, effectively extracting features from these regions and constructing a meaningful and clinically relevant prognostic risk score remains a significant challenge. Some approaches rely on manually designed features by physicians [28]: although these features often have strong clinical relevance, they are highly dependent on prior knowledge. This can lead to the overlooking of valuable information in pathological images. In contrast, some approaches depend entirely on deep features extracted by black-box deep learning models [29], which often lack interpretability. Although these models may perform well in a specific cohort, they are difficult to generalize to other cohorts, limiting their clinical applicability.

Based on the hypotheses derived from ATAT, the risk-scoring system we constructed contained information from three perspectives: 1) microscopic morphological features encoding the microstructures of necrosis, lymphocytes, and tumors; 2) co-localization features reflecting the spatial distribution and interactions between necrotic/lymphocytic and tumor regions; and 3) deep global features that incorporate overall information from WSIs. Among them, 1) and 2) consist of manually calculated and extracted features developed through ATAT, offering interpretability. To complement these interpretable manual features, we also utilized TransMIL to extract global features of the WSI. To integrate the features from these three views, we constructed a multi-view hybrid multiple instance learning framework and outputted HDS. Experimental results confirmed that for both OS and DFS, our HDS complements and refines existing clinical staging systems. Specifically, HDS demonstrated the ability to identify potential high-risk patients within the low-risk group (stratified by BCLC or TNM) in SYSUCC and TCGA-LIHC, thereby providing clinicians with valuable references to further adjust treatment intervention strategies for these patients.

Although this work achieved encouraging results in HCC prognostic analysis, it still has some limitations. For instance, the method of feature fusion from various perspectives employed in this study needs to be optimized. In fact, we are considering using manually extracted features to guide the extraction of global deep features in future work, which would make feature extraction more purposeful and further reduce the introduction of noise. On the other hand, for some HCC patients, we can only get their needle aspiration biopsy WSIs, which differ significantly in morphology from the WSI in our current cohort. Therefore, extending our approach to these patients presents a challenge at this stage. In the future, we plan to collect a more diverse set of external cohorts to broaden the applicability of our HDS.

## Conclusion

In conclusion, we developed a prognostic risk-scoring paradigm for HCC patients by drawing inspiration from the clinical diagnostic workflow of pathologists. This paradigm involves heuristically identifying potential high-risk tissue regions and constructing a multi-view hybrid risk score, offering new insights into how deep learning models can complement clinical staging systems of HCC patients. Experimental results indicate that HDS demonstrates promising performance in prognostic risk stratification and shows good generalizability to external cohorts, suggesting its potential as a valuable addition to existing clinical staging systems.

## Data Availability

The data that support the findings of this study are available from the Sun Yat-sen University Cancer Center Institutional Data Access/Ethics Committees, but restrictions apply to the availability of these data, which were used under license for the current study and so are not publicly available.

## Footnotes

1 https://portal.gdc.cancer.gov/projects/TCGA-LIHC.
